# Cushing syndrome in paediatric population: who and how to screen

**DOI:** 10.1007/s40618-024-02452-w

**Published:** 2024-09-30

**Authors:** Laura Chioma, Giuseppa Patti, Marco Cappa, Mohamad Maghnie

**Affiliations:** 1 Endocrinology and Diabetology Unit, Bambino Gesù Children’s Hospital, IRCCS, Rome, Italy; 2 Research Unit for Innovative Therapies in Endocrinopathies, Bambino Gesù Children’s Hospital, IRCCS, L.go Sant’Onofrio 4, Rome, 00165 Italy; 3 Department of Pediatrics, IRCCS Istituto Giannina Gaslini, Genova, Italy; 4 Department of Neuroscience, Rehabilitation, Ophthalmology, Genetics, Maternal and Child Health, University of Genova, Genova, Italy

**Keywords:** Cushing syndrome, Cushing disease, Diagnosis, Paediatric population

## Abstract

Cushing’s syndrome (CS) is characterised by signs and symptoms resulting from excessive and prolonged exposure to exogenous glucocorticoids or endogenous hypercortisolism. In childhood, exogenous CS represents the main cause of CS due to the widespread therapeutic use of glucocorticoids, while endogenous CS is very rare and accounts for about 10% of CS cases. According to the origin of the hypercortisolism, the ACTH-dependent form due to pituitary ACTH-secreting tumours is the most common form of endogenous CS in paediatric age (about 75–80% of cases), following by adrenal causes (about 15–20% of cases) including adenoma, carcinoma (which has a peak of incidence in the first decade), bilateral adrenal hyperplasia or Carney complex, with a different distribution by age. Ectopic ACTH-secreting CS, genetic forms of pituitary adenomas are more uncommon. The insidious onset of hypercortisolism and the absence of salient early signs make the diagnosis of endogenous CS difficult. Facial changes, weight gain with simultaneous growth failure, prepubertal virilisation, or hypogonadism in adolescence represent some of the key features of CS. The diagnostic workup is essentially aimed at confirming hypercortisolism through screening tests whose diagnostic accuracy is not 100% and therefore the combination of more than two tests is mandatory to confirm the diagnosis of CS.

## Introduction

Cushing’s syndrome (CS) may be defined as a clinical condition characterised by signs and symptoms resulting from excessive and prolonged exposure to glucocorticoids. CS can be differentiated into an exogenous form due to high-dose and prolonged glucocorticoid treatments and an endogenous form caused by excessive cortisol secretion.

In paediatric population, the exogenous CS represents the most frequent type of CS due to the widespread therapeutic use of glucocorticoids (given by systemic or local routes) for pulmonary, renal, haematological, or rheumatological diseases, more rarely due to an unappropriated administration of glucocorticoids by parents (medical child abuse or “Munchausen syndrome by proxy”). Endogenous CS is very rare, with an overall incidence of 1.2-5 per million per year [1–4], of which 10% of cases occurs in paediatric age [5, 6].

According to the origin of the hypercortisolism, endogenous CS can be also differentiated into an ACTH-dependent form resulting from ACTH-secreting pituitary neuroendocrine tumours (Cushing’s disease, CD) or ACTH-and or corticotropin releasing hormone (CRH) secreting neuroendocrine tumours outside the hypothalamic-pituitary area (ectopic Cushing syndrome, ECS), and an ACTH-independent form of adrenal origin (adrenal Cushing’s syndrome, ACS) (adenoma, carcinoma or bilateral adrenal hyperplasia). Finally, there are some clinical conditions, such as psychiatric disorders, severe obesity, poorly controlled diabetes mellitus, anorexia or intense physical exercise, that are associated with non-physiological hypercortisolism (non-neoplastic hypercortisolism, NNH, formerly known as Pseudo-Cushing’s syndrome) caused by chronic stimuli on hypothalamic-pituitary-adrenal axis.

NNH, particularly when characterized by moderate hypercortisolism, have often several clinical characteristics similar to CS and the first-line tests for screening endogenous hypercortisolism may provide misleading results, making the differential diagnosis very challenging. Besides the clinical history, the duration of symptoms and the first-line tests, second-line dynamic tests can be performed to better discriminate NNH from CS [7–9]. A recent systematic review and metanalysis provide an overview about the usefulness of the second-line tests to differentiate NNH from CS [10].

Similar to adult population, CD represents the most common form of CS in paediatric age (about 75–80%), while about 15–20% of cases are ascribed to ACS and less than 2% to ectopic origin, although there is a different distribution by age [11, 12]. In fact, CD occurs often in adolescent and pre-adolescent age, while endogenous CS in children younger than 8 years is mainly caused by adrenal tumours [13, 14]. CD in younger children with a relevant family history may be caused by rare genetic causes, since the pituitary adenomas should be the first presentation of MEN1, AIP gene mutations or more rare genetic mutations (as CDKNIB or DICER1 gene) [15].

According to some large epidemiological series studies, adrenocortical tumours present a peak incidence during the first decade, with a median age at diagnosis of 3–4 years [16] and are relatively more frequent in paediatric age than in adulthood. Paediatric adrenocortical tumours are almost always functional, presenting with virilization due to excess androgen secretion alone or in combination with hypercortisolism in about 80% of cases [16]. Adrenal tumours can be isolated or in the context of predisposing genetic syndromes as Li-Fraumeni or Beckwith-Wiedemann syndrome. Primary pigmented nodular adrenocortical disease (PPNAD) is a rare congenital disorder, occurring in late adolescence, mostly (about 95% of cases) associated with the multiple endocrine neoplasia (MEN) syndrome known as Carney complex [13]. Macronodular adrenal hyperplasia is rarely reported in the paediatric population, while another form of bilateral adrenocortical hyperplasia includes the adrenal lesions in McCune-Albright syndrome, which represents the first cause of CS in infants [5, 13, 17, 18]. ECS is extremely rare in childhood, and is associated to neuroendocrine tumours, mostly bronchial, thymic, renal and duodenal or pancreatic carcinoids [5, 13, 19, 20].

## Methods

An extensive MEDLINE search was performed in 2023 for the research question by two authors (LC, GP) independently, and discrepancies were resolved by discussion. A literature search was performed from 1970 to 2023. The following search words were included: “Cushing’s Syndrome, Cushing’s disease, children, childhood, diagnosis, endogenous hypercortisolism”. Search terms were linked to the Medical Subject Headings (MeSH) when possible. Keywords and free words were used simultaneously. Additional articles were identified with manual searches and included thorough review of other meta-analyses, review articles, and relevant references.

### Clinical presentation of CS in children

The diagnosis of CS is often difficult due to the insidious onset of hypercortisolism, in absence of relevant early signs of the disease, as well as the rarity of the disease in childhood. For these reasons, the time to diagnosis has been reported as a mean of 33 months (95% CI 29–38) and not dissimilar to adult population [21].

In childhood, the most common and earliest sign of CS is weight gain, which becomes pathognomonic when combined with concomitant growth failure. Generally, the discrepancy between height SDS and BMI SDS is suggestive of CS, although short stature (defined as height inferior to -2 SDS) is not always reported [22, 23]. On the other hand, decreased height velocity or growth arrest always occurs in childhood CS, due to the inhibitory action of glucocorticoids on growth plate cartilage, except for subjects presenting a concomitant hyperandrogenism in which growth may be normal or even increased. Some authors have suggested to consider children with height inferior to 0 SDS and BMI over + 1.5 SDS for CS diagnosis, allowing to differentiate from subjects with simple obesity, which often present tall stature [6, 24]. Ultimately, growth arrest could be considered the main red flag sign for paediatricians in suspected CS.

Other common signs reported in childhood and adolescence include swelling of the face (as plethora or moon face), headaches, *striae rubrae*, *acanthosis nigricans*, dorsal cervical or supraclavicular fat pads and osteopenia. The main clinical findings in paediatric CS are showed in Table 1.

**Table 1:** Clinical characteristics of paediatric cushing syndrome (CS)

	Magiakou 1994 [23]			Devoe 1997 [25]	Storr 2011 [26]	Shah 2011 [22]	Lonser 2013 [27]	Guemes 2016 [28]
Number of patients (F/M)	59 (37/22)			42 (25/17)	41 (15/26)	48 (19/29)	200 (106/94)	30 (14/16)
Period of observation	1982–1992			1974–1993	1983–2010	1988–2008	1982–2010	1983–2013
Subtype of CS	Pituitary (50)	Adrenal (6)	Ectopic (3)	Pituitary	Pituitary	Pituitary	Pituitary	Pituitary (16), Adrenal (11), Ectopic (2), Unknown (1)
Mean age at onset (y) or duration of symptoms (m)	11±4 y	9±6 y	10±3 y	NA	NA	23.6 ± 14.2 m	10.6 ± 3.6 y	12 m (6–18)
Mean age at diagnosis (range)	14±4	10±5	11±4	13.1 y (6.5–18)^a^	12.3 ± 3.5 y (5.7–17.8)	14.85 ± 2.5 y (9–19)	13.7 ± 3.7 y	8.9 (0.2–15.5)^a^
SDS Height at diagnosis (range)	-1.3±1.5	-1.0±1.3	-0.1±0.9	-1.8 (-3.5 to + 0.3)	-1.8 ± 1.3 (-1.2 to -4.2)	NA^b^	NA	-0.3 (-3.2 to + 3.0)^c^
**Signs and symptoms (%)**								
Weight gain	90			92	98	98	93	76.6
Growth retardation	83			84	100	83	63	36.6
Facial changes				46	100	98	63	
Fatigue	44			67	61		48	40
Pubertal lack or delay				60				10
Hirsutism	78			46	59		56	56.6
Acne	47			46	44		47	50
Amenorrhea (primary or secondary	78						49	
Virilization	38				76			26.6
Gynecomastia							16	
Osteopenia				74				
Dorsal cervical fat pad				28			69	
Striae rubrae	61			36	49	58	55	26.6
Acanthosis nigricans	12					75	32	
Headache				26	51		38	
Hypertension	47			63	49	71	36	50
Psychiatric disorders	19			44	59	46	31	43.3
Sleep disturbances								20
Muscle weakness						48		
Easy bruising				28		17	25	20
Glucose intolerance or diabetes						25	7	

Abbreviation. F: female; M: male; SDS: Standard deviation score; y: years; m: months; NA: not available. ^a^ median age; ^b^ 56% of subjects presented short stature; ^c^ median SDS height

The excess of adrenal androgens is responsible for the appearance of acne, hirsutism and early secondary sexual development (i.e., precocious pubic hair growth) in prepubertal children, while the consequent inhibition of gonadotropins secretion may lead of a lack or delay of pubertal development. However, in adolescence, adrenal hyperandrogenism and hypogonadism may result in menstrual changes (as oligo- or amenorrhea), virilization or gynecomastia. Adrenocortical tumours are often characterised by severe concomitant hyperandrogenism, presenting with hirsutism, acne or virilization.

Additional clinical features reported in paediatric population include depression, behaviour disorders (as anxiety, mood swings, emotional lability) and asthenia, while other typical signs of CS in adulthood as myopathy-related fatigue, easy bruising or hypertension are less common during childhood and adolescence [6, 22, 23, 25–29].

Considering the extreme rarity of CS and the increasing incidence of obesity in childhood, an extensive screening of the entire paediatric population with obesity is not recommended. It is however important to raise awareness amongst paediatricians to recognize few key features of CS, like facial changes, weight gain with simultaneous growth failure, prepubertal virilisation as menstrual changes or hypogonadism signs in adolescence.

Since the clinical features of NNH are often indistinguishable from neoplastic CS, a good history and examination (as individual growth charts), in addition to specific diagnostic tests, are needed to better rule out any physical or psychological causes of NNH [9].

In identify the different origin of CS based on symptoms, it should be considered that ECS is more commonly associated with catabolic signs (muscle weakness, osteoporotic fractures), little or no weight gain, hypertension and hypokalaemia due to the mineralocorticoid effect of cortisol excess. In fact, very high cortisol levels can cause the saturation of the type-2 11β-Hydroxysteroid Dehydrogenase (11βHSD-2) enzyme, expressed in renal cortex and responsive to convert cortisol into inactive cortisone, leading to spillover of cortisol to the mineralocorticoid receptor. Because of this biochemical mechanism, severe hypercortisolism may be considered as a functional mineralocorticoid excess state causing hypokalaemia, increased renal tubular sodium reabsorption, consequent intravascular volume expansion and hypertension [30–32]. However, since the clinical spectrum of presentation of ECS may overlap with CD, the differential diagnosis is challenging and requires the combination of dynamic biochemical testing and multimodal imaging, each with its own pitfalls [17, 20, 33].

### Diagnostic workup for CS

Once a possible intake of exogenous corticosteroids has been ruled out through a careful medical history, the first step in the diagnostic workup is the identification of endogenous hypercortisolism.

## Screening for endogenous hypercortisolism

Endogenous hypercortisolism in the paediatric population is essentially demonstrated with the following tests: 24-h urinary free cortisol (UFC), late-night salivary or serum cortisol and dexamethasone-suppression testing. Because none of these tests has 100% of diagnostic accuracy, as for adulthood, at least two tests are usually needed to confirm endogenous CS [7]. Table 2 shows the statistical features of the three diagnostic tests reported in the paediatric population.

**Table 2:** Diagnostic tests performed for endogenous hypercortisolism screening in the paediatric population

Author	Population Age (mean)	Subject characteristics (*N*)	Test	Cut-off	Sensibility	Specificity
Bickler 1994 [54]	15.7 y (pituitary) 8.1 y (adrenal)	Pituitary (10) Adrenal (2)	UFC	> 60 mg/m^2^	100% (8/8)	
			LDDST	< 50% of basal serum cortisol	91% (10/11)	
Devoe 1997 [25]	13.1 y (6.5–18)^a^	Pituitary (42)	UFC	> 70 µg/m^2^	86% (25/29)	
Martinelli 1999 [49]	10.2 ± 5 y	Pituitary (5), Adrenal (6), Obese controls (21)	Late-night salivary cortisol	> 7.5 nmol/l	100% (11/11)	95.2% (20/21)
Gafni 2000 [39]	5–17 y	CS patients (14), Healthy controls (53)	UFC	> 72 µg/m^2^	93% (13/14)	100% (53/53)
			Late-night salivary cortisol	> 7.5 nmol/l	93% (13/14)	100% (53/53)
Davies 2005 [47]	12.2 y	Pituitary (14)	Late-night serum cortisol	> 50 nmol/l [1.8 µg/dl]	100% (14/14)	
Batista 2007 [38]	3–18 y	Pituitary (80), Adrenal (25), Controls (20)	UFC	> 70 µg/m^2^	88% (92/105) [PPV 98%]	90% (18/20) [NPV 58%]
			Late-night serum cortisol	> 4.4 µg/dl	99% (104/105) [PPV 100%]	100% (20/20) [NPV 95%]
Shah 2011 [22]	14.85 ± 2.5 y	Pituitary (48)	Late-night serum cortisol	> 3.2 µg/dl	100% (38/38)	
			LDDST (30 µg/kg/day [max 2 mg/day] divided every 6 h for 48 h	≥ 1.8 µg/dl	100% (48/48)	
				≥ 5 µg/dl	94% (45/48)	
Storr 2011 [26]	12.3 ± 3.5 y	Pituitary (41)	LDDST (30 µg/kg/day [max 2 mg/day] divided every 6 h for 48 h)	< 50 nmol/l [1.8 µg/dl]	92% (35/38)	
Lonser 2013 [27]	13.7 ± 3.7 y	Pituitary (200)	UFC	Age-appropriate reference	99% (177/179)	
				> 70 µg/m^2^	88% (155/177)	
			Late-night serum cortisol	> 7.5 µg/dl	97% (188/193)	
Shapiro 2016 [40]	11.7 y (pituitary), 12.9 y (adrenal), 11.5 y (controls)	Pituitary (39), Adrenal (8), Control (19)	UFC (different assays)	Corrected for BSA	89% (34/38)	100%
Wędrychowicz 2019 [55]	11.7 y	Pituitary (4)	UFC	> 55 µg/24 h	100% (4/4)	
			Late-night serum cortisol	> 4.4 µg/dl	100% (4/4)	
			Overnight DST (1 mg at 11.00 p.m.)	< 1.8 µg/dl	75% (3/4)	
Guemes 2016 [28]	8.9 y (0.2–15.5)^a^	Pituitary (16), Adrenal (11), Ectopic (2), Unknown (1)	UFC	> 275 nmol [100 µg]/24 h	94% (17/18)	
			Late-night serum cortisol	> 138 nmol/l [5 µg/dl]	100% (27/27)	
			LDDST (20 µg/kg/day [max 2 mg/day] divided every 6 h for 48 h)	< 50 nmol/l [1.8 µg/dl]	100% (20/20)	

Abbreviation. N: number; y: years; UFC: Urinary free cortisol; DST: dexamethasone suppression test; LDDST: low-dose DST; PPV: Positive Predictive Value; NPV: Negative Predictive Value; BSA: body surface area. ^a^ median age

Recently, some authors have reported the value of hair cortisol measurements as a good marker of hypercortisolism also in paediatric population [34], although further studies are needed to validate this test in the diagnostic workup for CS.

### 24-h Urinary free cortisol (UFC)

24-h UFC is a long-time used screening test for CS, widely performed in childhood for its non-invasive characteristics and the possibility to collect the 24-h samples at home, although this collection may be difficult for younger subjects. Differently from adults, in paediatric population UFC should be corrected for body surface area, conventionally used to make the normal range homogeneous despite the different cortisol secretion during childhood and puberty [35–37]. The cut-off of 70 µg/m^2^/day is associated with an acceptable sensitivity and specificity (over 88% and 90% respectively) [27, 38, 39], even if the normal ranges varied among different paediatric studies, due to assay-specific reference range [25, 28, 40]. In order to reduce intra-patient variability and to provide a better diagnostic accuracy, it is now recognised that at least two UFC measurements should be performed in subjects suspected of CS [27, 38, 40, 41].

Mild forms of hypercortisolism may have a false-negative UFC assay, because free cortisol appears in the urine only when serum cortisol exceeds the plasma protein binding capacity. On the other hand, false-positive elevation of UFC measurements should be caused by NNH, as physical or emotional stress, severe obesity or depression. In fact, obese children and adolescents may present slightly elevated UFC, particularly when the obesity is associated with metabolic syndrome [42, 43].

Considering the extremely low prevalence of CS in the paediatric population, the positive predictive value of UFC measurements is considerably low. For this reason, UFC alone is not recognised as an ideal screening tool, while its use combined with another screening tests is desirable to better detect subjects with endogenous hypercortisolism.

In the last decades, liquid chromatography-tandem mass spectrometry (LC-MS/MS) assays had demonstrated superior sensitivity and specificity compared to traditional immunoassays [44–46], reducing a considerable analytical bias thanks to its ability to differentiate various glucocorticoid metabolites.

### Late night cortisol

Abnormal circadian rhythm of cortisol secretion is a hallmark of CS. The lack of the physiological evening nadir in cortisol secretion is detectable with late-night serum or salivary cortisol tests. As for UFC, at least two late-night cortisol measurements are desirable to improve the diagnostic accuracy, particularly in patients with mild CS.

For serum cortisol measurement, an indwelling intravenous cannula should be placed before sleeping and the blood sample should be taken without waking the child. The assessment of midnight serum cortisol gives the highest sensitivity and specificity for the diagnosis of CS in childhood (99 and 100% respectively using the cut-off of 4.4 µg/dl) [38], despite different normal ranges (between 1.8 and 5 µg/dl) have been considered for paediatric subjects [22, 28, 47]. However, the late-night serum sample requires hospitalization and its use as a screening test for CS is limited.

On the other hand, late-night salivary cortisol measurement represents an easily executable, stress-free test also in outpatient setting. Conventionally, the salivary samples are collected at 11–12 pm, even if some authors suggest to performed it at usual bedtime in order to achieve unstressed levels, resulting from the request to the patient to stay awake beyond the usual bedtime [48]. This precaution, suggested for adult subjects, should be considered also for paediatric population to reduce a potential false-positive rate of the test.

Although the available data in paediatric population are limited, the sensitivity and specificity of late-night salivary cortisol assessment appear to be close to late-night serum cortisol (93–100% and 95–100% respectively) [39, 49].

For all these reasons, late-night salivary cortisol seems to be the best screening test for endogenous hypercortisolism in childhood.

Although the traditional immunoassay methods already have a very high sensitivity, LC-MS/MS assays had demonstrated an improvement of diagnostic specificity and appear to be the most accurate analytical tools also for modern salivary or serum steroid measurements [50–52]. In fact, the use of LC-MS/MS assay allows the dosage of different cortisol metabolites (as cortisone) in order to better identify the endogenous cortisol production and consequently to reduce false-positive results [8, 51].

### Low-dose dexamethasone suppression tests (DST)

In healthy individuals, a supraphysiological exogenous dexamethasone dose inhibits ACTH and consequently cortisol secretion. Therefore, a decrease of serum cortisol concentration below the value of 1.8 µg/dl after 1 or 2 mg dexamethasone dose is considered to be a normal response. The low-dose DST should be performed through two different forms: the 1 mg “overnight” (or Nugent) and the two-day 2 mg (or low-dose Liddle) test.

The “overnight” DST is performed with the administration of 1 mg (or 25 µg/kg in children with body weight < 40 kg) of dexamethasone at 11 PM to 12 AM (midnight), measuring serum cortisol at 8 AM the next morning. In order to ensure a proper DST in adult population, Ceccato et al. propose to measure also dexamethasone after 1 mg-DST with LC-MS/MS assay [53]. At present, no similar data are available among paediatric population, although dexamethasone measurement should be suggested also in children and adolescents to reduce false-positive results due to inadequate bioavailability or incorrect administration of dexamethasone.

The “low-dose Liddle” DST (LDDST) consists of the administration of 2 mg/day of dexamethasone (or 20–30 µg/kg/day in children < 30 kg), divided in 0.5 mg doses every six hours for 48 h, and measurement of serum cortisol within six hours after the last dose.

For both DST, the lack of the physiological serum cortisol suppression (< 1.8 µg/dL) is suspicious for CS. LDDST has demonstrated a good sensitivity (over 90%) for CS in paediatric patients [26, 28], whereas less data regarding the overnight DST sensitivity and specificity are available in childhood [54, 55].

For its ease analysis in an out-patient setting, LDDST is therefore a useful screening test for paediatric patients suspected of CS.

Recently, some authors have investigated the utility of salivary cortisone measurement after DST, that is characterized by a more linear relationship with serum cortisol than salivary cortisol [56]. Moreover, a prospective use of salivary cortisol/cortisone after DST in childhood should be encouraged for its non-invasive and stress-free peculiarity, avoiding venipuncture.

## Etiological diagnosis of endogenous CS

### Basal electrolytes and ACTH

Levels of serum electrolytes are usually normal, but potassium may be decreased, especially in children with ECS [57]. In children with CD, morning plasma ACTH is commonly detectable (> 5 pg/ml) while those with ACS showed suppressed ACTH [29]. Batista et al. showed that a cut-off of morning ACTH of 29 pg/ml had a sensitivity of 70% and specificity of 100% to differentiate ACTH-dependent from ACTH-independent CS [38]. ACTH concentrations are usually very high in patients with ECS but may be normal in patients with pituitary adenomas [17, 29, 57]. CD should be suspected in patients with biologically moderate signs, without hypokalaemia or marked plasma ACTH elevation and with progressive onset [17, 20, 33].

### CRH stimulation test

The CRH test has been suggested as the best non-invasive tool for diagnosing CD. Sensitivity and specificity are reported to be around 80 and 92% (according to study in adults) [17, 58–60]. This test consists in the intravenous injection of 1 µg/kg CRH (maximum dose 100 µg) [29]. The criterion for diagnosis of CD is a mean increase of 20% above baseline for cortisol value at 15 and 30 min and an increase in the mean ACTH concentration of at least 35% over basal value at 15 and 30 min after CRH administration [17, 29]. Some authors reported the use of ovine CRH (the only available form in the United States, until the mid-2020) in paediatric population [38, 61] as alternative to human CRH. Although it has been described as the ovine CRH can induce a stronger, more prolonged increase in ACTH and, particularly, cortisol compared with human CRH in adult subjects [62], no data are available comparing ovine and human CRH in paediatric population.

Despite children with CD seem to have a more evident cortisol response than adults, making this test more useful in the paediatric age than in adults [17, 26, 29, 63], the recent synthetic human CRH shortage [64] will make CRH test less feasible in favour of other dynamic tests as Desmopressin test [65].

### Desmopressin test

Desmopressin is a preferential vasopressin receptor V2 and V3 agonist. Because of the overexpression of the V3 in human ACTH-secreting adenomas, the administration of desmopressin causes a significant rise in ACTH and cortisol levels in most patients with CD [17, 58]. This makes desmopressin administration a suitable test enabling the distinction between neoplastic from NNH [9, 10, 26]. Like CRH test, Desmopressin test results effective, well-tolerated, less expensive, and relatively non-invasive. While the sensitivity is comparable to CRH test, the specificity seems to be lower [17, 58, 60, 66]. Like the other tests, it is probabilistic: the more significant the elevation of ACTH and cortisol, the more probable the diagnosis of corticotropic adenoma [17, 58]. Different cut-off criteria were used to define a positive response. Malerbi et al. showed that the administration of Desmopressin 5–10 µg intravenous determines a cortisol increase above baseline ranging from 61 to 379% in patients with pituitary disease [67]. Sakai et al. using a high percent ACTH rise threshold of 120% reported a positive ACTH response in all 10 patients with CD, whereas all 3 patients with ECS were unresponsive to desmopressin [68]. Tsagarakis et al. showed that desmopressin test (10 µg intravenous) can produce a significant overlap of responses between CD and patients with ECS and therefore it is of limited value in the differential diagnosis of ACTH-dependent CS. This is probably due to the expression of the V2 receptors in tumours with ECS [69]. Desmopressin (10 µg intravenous) in combination with CRH may provide an improvement over the standard CRH test in the differential diagnosis of ACTH-dependent CS [70]. However, the benefit of a desmopressin-CRH combined test results limited [66]. It should be considered that all the above studies included adults [67–69]. Desmopressin test proved to be effective in increasing the sensibility of Bilateral Petrosal Sinus Sampling (BIPSS) [71]. In a retrospective study including 16 children with CD, Chen et al. showed an increase of the sensitivity of BIPSS from 64.7% at baseline to 83.3% after desmopressin stimulation [72]. Many CD patients respond aberrantly to the desmopressin test. Loss of the desmopressin response, performed in the early post-operative period, is a good predictor for a favourable long-term outcome. Moreover, during follow-up, the return of desmopressin response is predictive of recurrence [66, 71].

### Standard high dose dexamethasone suppression test (HDDST)

HDDST or high-dose Liddle test is the oldest described and it is used to differentiate CD from ECS. This test consists in the administration of dexamethasone at a dosage of 80–120µgr/kg/day divided into four doses every 6 h (maximum 2 mg/dose) for 48 h or a single cumulative dose of 80–120µgr/kg (maximum 8 mg) at 11 pm. Plasma cortisol is measured at 8–9 am the morning after the last administration of dexamethasone; the suppression of serum cortisol up to 50% of baseline is suspicious for CD as for adult population [17, 26, 28, 29, 38].

Liu et al. showed that HDDST in combination with pituitary dynamic enhanced MRI (dMRI) had a positive predictive value (98.6%), higher than that of Bilateral Petrosal Sinus Sampling (BIPSS) for the diagnosis of CD [73].

Despite HDDST had reported a good sensibility to identify CD in childhood, this test seems to have a low specificity to exclude ECS because of the high degrees of cortisol suppression after HDDST in children with ECS [19, 28, 29]. In addition, the administration of high-dose dexamethasone in CS patients with high cortisol level can cause severe side effects, including exacerbation of their hypertension and fluctuation of blood glucose. Because of the low accuracy and the risk of severe side effects, this test is less frequently used [29].

**Fig. 1:**
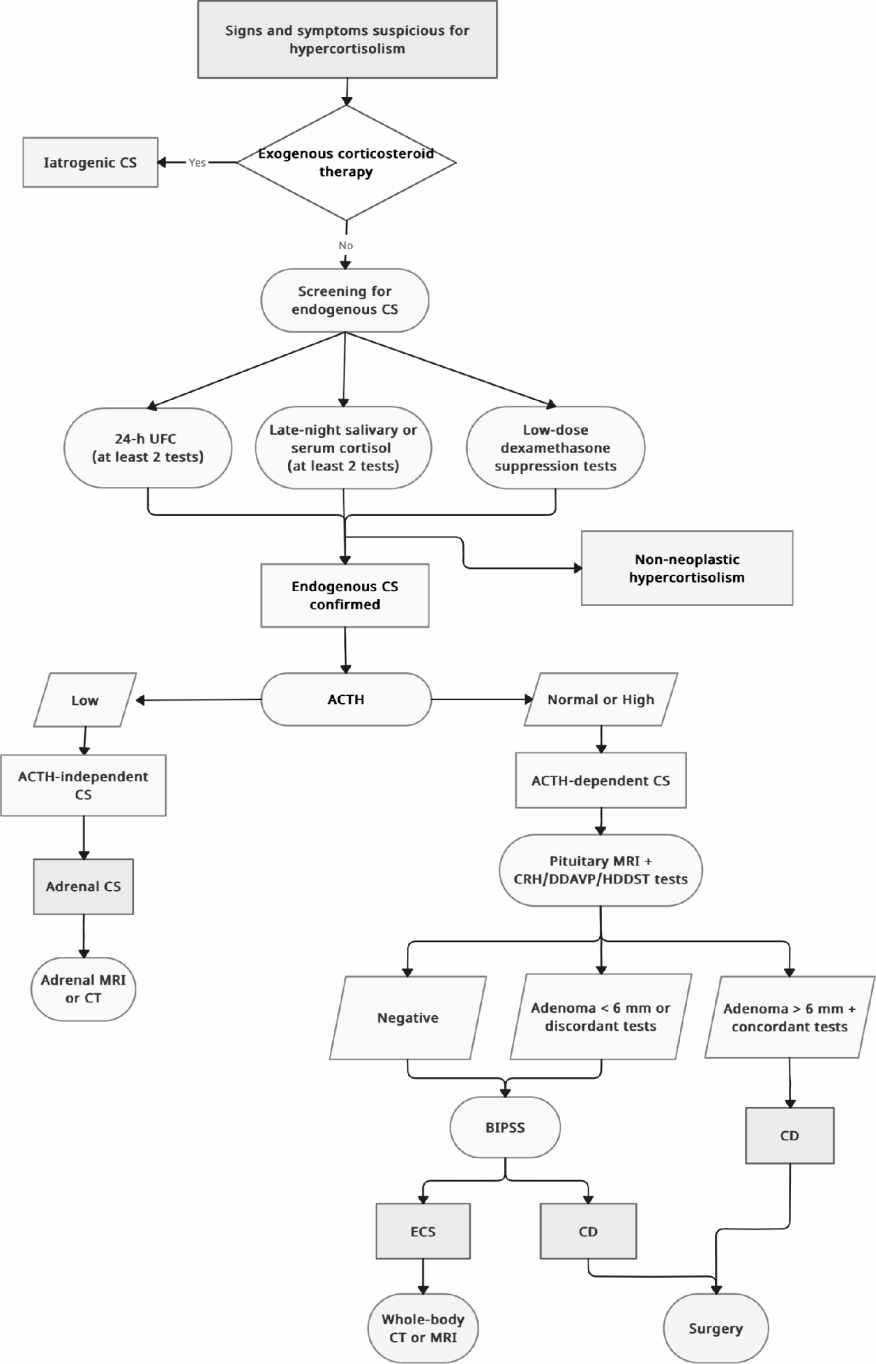
Diagnostic algorithm for screening and differential diagnosis of cushing syndrome in paediatric population

### Imaging

#### Pituitary magnetic resonance imaging (MRI)

Since ACTH-secreting pituitary adenomas are very small (usually < 6 mm in diameter), it is difficult to localize these tumours. Diagnostic workup of CD includes pituitary MRI, but in many patients no tumour is identified. Conventional MRI, even with contrast enhancement, mostly failed to identify ACTH-secreting microadenomas in children with CD. Up to one-third of paediatric and adolescent patients with CD don’t have pituitary tumour detectable at brain MRI. The acquisition protocol should comprise coronal and sagittal spin-echo (SE) slices with gadolinium-enhanced T1 and T2 and millimetric 3D T1 slices [17, 29, 57, 74]. In a retrospective study including 30 children with CD (mean age 12 ± 3 years), Batista et al. showed that pre- and post-contrast spoiled gradient-recalled acquisition in the steady state (SPGR) was superior to conventional pre- and post-contrast T1-weighted SE acquisition MRI in the identification of the microadenomas. In particular, the post-contrast SPGR-MRI identified the location of the tumour in 18 of 28 patients, whereas post-contrast SE-MRI identified the location and accurately estimated the size of the tumour in only 5 of 28 patients (*p* < 0.001) [74].

#### Bilateral petrosal sinus sampling (BIPSS)

BIPSS is another powerful diagnostic tool with high sensitivity and specificity, but its invasiveness and high cost limit its wide application, and the indication for BIPSS is still controversial [7, 17, 29, 75, 76]. It consists of the placement of femoral catheters that reach the inferior petrosal sinuses. Successively, blood samples are collected for measurement of ACTH from petrosal sinuses and from peripheral pathway before and after the administration of CRH. Inferior petrosal sinus (IPS) to peripheral (P) ACTH ratio and interpetrosal sinus gradient of one of the two sides to the contralateral side are calculated [75, 76]. In order to avoid incorrect results, it is recommended to verify hypercortisolism with serum cortisol sample immediately before performing BIPSS. Detomas et al. recently described the largest study on BIPSS. According to the authors, the cut-offs for the ACTH IPS: *P* ≥ 1.9 at baseline (sensitivity 82.1%, specificity 85.7%) and ≥ 2.1 at 5 min post-CRH (sensitivity 91.3%, specificity 92.9%) allow for the best discrimination between CD and ECS [77]. In a multicentre study including 16 children aged between 4 and 16.5 years, Turan et al. showed that BIPSS is a superior diagnostic work-up than MRI to confirm the diagnosis of CD. Moreover, it showed a significantly higher sensitivity (92.8%) than MRI (53.3%) in detecting adenoma localization at pituitary level, which is crucial for surgical intervention [75]. The use of desmopressin has been reported in alternative to CRH [76]. In a review including case series of children with CS [76], the overall accuracy of BIPSS was 84.1% and became 92.3% after stimulation with desmopressin. The overall lateralizing accuracy of BIPSS was 50%. While BIPSS has a high diagnostic accuracy for the localization to the pituitary gland, it is not reliable for tumour lateralization to the right or left side of the gland. BIPSS is considered the gold standard to reliably exclude ECS and should performed in a specialized centre due to potential patient risk. However, BIPSS is not routinely available in many centres, it may have decreased specificity in children, especially when the pituitary tumour is not lateralized showing misleading results [77, 78]. For these reasons and for the risks related to the invasiveness of the procedure, BIPSS should be reserved only for exceptional cases in children [17, 75, 76].

#### Radiological anatomic imaging

Subjects with ACS should perform an adrenal Computer Tomography (TC) or MRI to determine the adrenal cause. Despite abdominal TC with contrast-enhanced studies is the cornerstone of imaging of adrenal tumours in adults, MRI scan should be initially preferred in childhood to avoid radiation exposure [79]. Adrenocortical carcinomas are usually unilateral, larger than adenomas, with irregular margins, inhomogeneous contents (with areas of necrosis, haemorrhage and calcification) and avidly enhancement after contrast administration due to their high vascularity [80]. PPNAD is more difficult di identify with radiological studies, because it usually presents normal- or small-sized adrenal glands.

In subjects with suspected ECS, a thin-multislice neck-chest-abdomen-pelvic CT, alone or eventually followed by MRI, should be performed to identify neuroendocrine tumours that generally are very small and difficult to identify [11].

#### Functional imaging

Second-line functional imaging studies (as Positron Emission Tomography, PET, or scintigraphy) may be useful to provide an accurate etiological diagnosis of CS, particularly when the traditional radiological exams are inconclusive to differentiate CD from ECS. Because of the rarity of ECS, a univocal algorithm regarding the use of new molecular imaging techniques is not well established.

Whereas the ectopic ACTH-secreting tumours express the cell-surface receptors for somatostatin, ^111^In-pentetreotide (OCT) scintigraphy is often chosen as confirmatory exam [81].

The ^68^Gallium-DOTATATE PET/CT scan, using a modified octreotide molecule that also binds to somatostatin receptors, has shown a greater sensitivity for small tumours and may be useful for the tumoral identification in case of negative OCT scan [7]. Finally, ^18^FDG-PET/CT seems to be highly sensitive for the detection of aggressive pancreatic lesions [81].

In ACS cases, when adrenocortical carcinoma is suspected and traditional imaging studies (MRI or TC) are not diriment, ^11^C-metomidate-PET/CT scan allows a non-invasive characterization and staging of the adrenal lesion [82, 83].

## Algorithm approach

Clinical history and the age at presentation of symptoms should guide throughout the different diagnosis of endogenous CS. A careful personal history, supported by patient growth charts, physical examination and screening tests should be able to rule out any physical or neuropsychiatric causes of NNH, even if second-line dynamic tests are sometimes needed to distinguish NNH from neoplastic CS.

Although CD is the main cause of CS in children older than 8 years, the clinical presentation of ECS may overlap with CD and the differential diagnosis of CS may be challenging, requiring the combination of dynamic biochemical tests and multimodal imaging.

Since none of the dynamic tests show a perfect sensitivity and specificity, using more than one dynamic test might improve accuracy. A non-invasive approach using a combination of three or four tests, specifically CRH and desmopressin stimulation tests plus MRI, followed by total-body CT, if biochemical and anatomical findings are discordant, correctly diagnose CD in approximately half of patients, potentially eliminating the need for BIPSS [17, 84]. If a pituitary tumour is detected on MRI and dynamic testing results are consistent with CD, BIPSS is not necessary for diagnosis. Since ECS in children is extremely rare, the algorithm approach in children may differ from the adult approach. Findings of ACTH-dependent CS, doubtful CRH test and normal pituitary MRI should be followed by extended imaging (whole-body CT/MRI or functional imaging). Considering the extremely rarity of ECS, the great majority of ACTH-dependent hypercortisolism, even with normal pituitary MRI, corresponds to CD due to a pituitary lesion not yet visible [17]. For this reason, BIPSS should be used only exceptionally in children. A diagnostic algorithm is proposed in Fig. 1.

## Conclusions

We provide detailed revision on the diagnostic evaluation of children and adolescents presenting with signs and symptoms suspicious for CS and guidance on the workup from the confirmation of endogenous hypercortisolism to the etiological diagnosis of such a rare challenging condition.

## Data Availability

Not applicable.

## Abbreviations

CS: Cushing syndrome
CD: Cushing disease
ECS: ectopic ACTH-dependent CS
24-h UFC: 24-h urinary free cortisol
DDAVP: Desmopressin
CRH: corticotropin releasing hormone
HDDST: High Dose Dexamethasone Suppression Test
MRI: Magnetic resonance imaging
CT: computed tomography;
BIPPS: Bilateral Petrosal Sinus Sampling
