# Correction: Cushing syndrome in paediatric population: who and how to screen

**DOI:** 10.1007/s40618-024-02485-1

**Published:** 2024-11-09

**Authors:** Laura Chioma, Giuseppa Patti, Marco Cappa, Mohamad Maghnie

**Affiliations:** 1 Endocrinology and Diabetology Unit, Bambino Gesù Children’s Hospital, IRCCS, Rome, Italy; 2 Research Unit for Innovative Therapies in Endocrinopathies, Bambino Gesù Children’s Hospital, IRCCS, L.go Sant’Onofrio 4, Rome, 00165 Italy; 3 Department of Pediatrics, IRCCS Istituto Giannina Gaslini, Genova, Italy; 4 Department of Neuroscience, Rehabilitation, Ophthalmology, Genetics, Maternal and Child Health, University of Genova, Genova, Italy

**Correction: Journal of Endocrinological Investigation** 10.1007/s40618-024-02452-w

In the original version of this article, some part of the Funding information was missing. The funding information was previously read “This research did not receive any specific grant from any funding agency in the public, commercial or not-for-profit sector” Should have been “This research did not receive any specific grant from any funding agency in the public, commercial or not-for-profit sector. Open Access funding for this article was provided by Recordati Rare Diseases Italy S.r.l. The funder had no role in the preparation, review, or approval of the manuscript.”

The original article has been corrected.

